# The mechanomyographic dataset of hand gestures harvested using an accelerometer and gyroscope

**DOI:** 10.1016/j.dib.2025.111558

**Published:** 2025-04-14

**Authors:** Khalid A. Abbas, Mofeed Turky Rashid, Luigi Fortuna

**Affiliations:** a Electrical Engineering Department, University of Basrah, Basrah, Iraq; b Computer Engineering Department, University of Basrah, Basrah, Iraq; c Dipartimento di Ingegneria Elettrica Elettronica e Informatica

**Keywords:** Biomedical engineering, Biomedical signal processing, Human-computer interaction, Gesture recognition, Classification, Mechanomyogram, Accelerometer, Gyroscope

## Abstract

Mechanomyography (MMG) datasets are crucial due to their unique characteristics, non-invasive techniques, fewer required sensors, improved signal-to-noise ratio, lightweight equipment, and no need for skin preparation, unlike some other techniques. This paper introduces a mechanomyography (MMG) signal dataset intended for application in human-computer interaction (HCI) research. The dataset is obtained from integrated sensor data, capturing mechanical signals from muscle activity via the accelerometer, augmented by the gyroscope for motion analysis. The dataset comprises 6-axis accelerometer and gyroscope data from 43 participants, ranging in age from 18 to 69 years, exhibiting a male-to-female distribution of 60 % to 40 % respectively. The dataset includes the following 11 gestures: clapping, coin flipping, finger snapping, fist making, horizontal wrist extension, index finger flicking, index thumb tapping, shooting, thumb up, wrist extension, and wrist flexion. A novel, assembled, and manufactured wearable system collected data from the main muscles that end at the wrist, just below the watch strap. These muscles include flexors and extensors, which work together to move the wrist and fingers when making the hand gestures listed above. Every participant completed a total of fifty repetitions for each of the eleven hand motions, resulting in 550 samples per subject. Before recording the signals, a demographic survey with the participants is conducted. Researchers focusing on classification, recognition, and prediction can use the gathered data to develop MMG-based hand motion controller systems. The collected data can also serve as a reference for developing a model using artificial intelligence (e.g., a deep learning or machine learning model) that is capable of identifying gesture-related MMG signals. It is suggested that the proposed dataset is used to evaluate existing datasets in the literature or to validate artificial intelligence models developed with alternative datasets through the participant-independent evaluation approach. This dataset can be useful in a variety of applications and fields, including interaction between humans and robots, gaming, assistive technology, healthcare observation, and sports analytics, to name a few specific examples.

Specifications Table

**Table:** 

Subject	Engineering > Biomedical Engineering] [Computer Science > Human-Computer Interaction
Specific subject area	Signal processing, classification of MMG signals, recognition of hand gestures, and gaming.
Type of data	Filtered, Table (.csv, .mat)
Data collection	The MMG measurements were obtained with the MPU-92/65 sensor module's tri-axial accelerometer and tri-axial gyroscope. This module is designed to be worn like a watch, facilitating effortless and seamless usage. This module is linked to the initial ESP32 DEVKIT V1 microcontroller, which functions as a transmitter, transmitting samples of gesture data to the receiver using Wi-Fi. The receiver is the second ESP32 microcontroller of the same type, which is directly linked to the laptop with a USB cable. The six-axis raw data for each executed gesture were acquired at a sampling frequency of 200 Hz by the first ESP32 microcontroller, which relays the data to the laptop through the secondary ESP32 microcontroller over a Wi-Fi connection. The data is further processed using a 4th order Butterworth bandpass filter with a frequency range of 30-90 Hz to remove motion artifacts and high-frequency noises. Prior to initiating the recording of signals for the gestures under investigation, each participant was asked a series of questions. The objective of these inquiries was to collect background information on each participant and do a demographic evaluation. This was executed to identify the suitable individuals for gathering the dataset, as specified in Table 2. Subsequently, a 30-to-45 minute specialized training session was administered for each participant, elucidating the study's objectives and purpose while exhibiting all gestures in their presence, accompanied by a comprehensive explanation of their implementation. The MMG signals data were gathered from the dominant hand of each participant.
Data source location	[In order to implement a more realistic application circumstance, the samples were collected in a diverse array of environments, such as the laboratories of the College of Engineering at the University of Basrah, residence, employee office, and gym in Basrah city, Iraq.]
Data accessibility	Repository name: Mendeley Data Data identification number: 10.17632/mkhn7kxjvy.3 Direct URL to data: 10.17632/mkhn7kxjvy.3 https://data.mendeley.com/datasets/mkhn7kxjvy/3
	Download All direct URL: https://prod-dcd-datasets-cache-zipfiles.s3.eu-west-1.amazonaws.com/mkhn7kxjvy-3.zipSHA-256 checksum: “bbd2d745b64f3ce43ab353f7ffab347b91c16e626ce1a63dcfc8e34dbee84880”
Related research article	none

## 1 Value of the Data

• Dataset of this article comprises a substantial collection of data from 43 people. The forty-three participants exhibit age diversity, spanning from 18 to 69 years; gender balance, with a distribution of 60 % male and 40 % female; and physical activity levels, comprising 35 % athletic and 65 % non-athletic individuals. This composition facilitates subsequent studies to analyze variations among age groups, gender, or physical activity status of the participants.
• Each participant executed 11 hand motions, and the data from each repetition comprised a 6-axis time series of the MMG signal, incorporating the 3-axis data from both the accelerometer and gyroscope. The dataset contains 23,650 six-dimensional gesture samples, which were recorded at a rate of 550 samples per subject after 50 repetitions of each of the eleven motions used in this work (11×50×43 = 23,650). Each gesture sample comprised six directions of time series signals, with two signals for each of the x, y, and z axes from the accelerometer and gyroscope sensors. As a result, this dataset has a total content of 141,900 signals (23,650×6 = 141,900). Thus, this can be a great asset for demanding large-scale data sets, such those used to build machine learning and deep learning models and as a reference dataset, it helps models be benchmarked.
• The data that is provided is filtered to eliminate other sources of noise, allowing the researchers to concentrate solely on the muscle-generated noiseless signals. It is important to note that the filter that was applied to the raw data was determined and chosen after conducting numerous experiments and modifications to its parameters in relation to its type, degree, and frequencies.
• The dataset has been uploaded and made available online in two different hierarchical structures. The first structure organizes the data based on the gesture name, while the second structure organizes the data based on the subject number. This arrangement increases the potential benefit and ease of handling, allowing for the possibility of reassembling the data in multiple ways as needed.
• To align with the practical realities of potential applications for this data, creating an ideal environment for signal acquisition from participants was deliberately avoided. Instead, various atmospheres tailored to each participant's preferences were selected, ensuring their comfort and authenticity during signal collection, even if they wished to perform their gestures in multiple locations.
• The biomedical signal engineering societies will find the data to be particularly beneficial for the duties of hand-gesture recognition and classification, which can be applied in HCI applications.
• This data is valuable for the development of artificial intelligence algorithms, applicable in both machine learning and deep learning research, for predicting hand motions. This set of data works well for training, validating, and testing novel hand gesture recognition systems based on accelerometer and gyroscope data, as well as testing by presenting unseen data to previously trained and validated systems for generalization.
• The significant application resides in human-robot interaction. The dataset facilitates the advancement of sophisticated gesture recognition systems that enhance human-robot interaction. Facilitating robots' comprehension and reaction to human gestures might yield more natural and seamless interactions in sectors such as manufacturing, healthcare, and personal support.
• The gaming sector can get substantial advantages from our dataset. It can enable the execution of gesture-based controls, facilitating fully immersive gaming experiences. Integrating gesture identification into gaming systems enables developers to build more realistic and interactive experiences that dynamically adapt to user movements.
• The dataset holds promise for advancing the creation of assistance devices. These apparatuses utilize gesture recognition to effectively respond to user inputs, significantly improving accessibility for people with disabilities. By offering a method of control via gestures, we enable users to engage with technology in a more efficient manner.
• Applications for healthcare monitoring can make use of the dataset. It can help with patient mobility tracking, which is important for fall detection technologies and rehabilitation procedures. Healthcare providers can learn more about a patient's progress and make sure that rehabilitation plans are customized to meet each patient's needs by evaluating motion data.
• A notable domain is sports analytics, when the dataset can be employed to examine athletes' moves. This study may facilitate the formulation of enhanced training routines and efficacious injury prevention techniques. By analyzing the intricacies of motion recorded by accelerometers and gyroscopes, athletic trainers and coaches can make informed judgments to improve performance and mitigate injury risk.
• Rehabilitating, gaming purposes, and applications for smart homes are just a few of the many valuable fields that can benefit from the extensive gestures included in the dataset we offer. In interactive situations, the clapping gesture can be used to communicate attention, while in gaming, the fist-making and coin-flipping gestures can be used for decision-making and action commands. Users can improve their control over assistive technologies and accomplish tasks with more accuracy by using wrist movements, such as flexing and horizontally extending the wrist. For even better mobile device engagement, touch-based interfaces rely heavily on index finger actions like flicking and tapping. By making this information available, we are encouraging academics and developers to build better gesture detection algorithms, which will improve accessibility and the user experience.

## 2 Background

Hand gesture categorization is a pivotal research area for gesture-based systems that significantly enhances the interaction between humans and computers. The integration of HCI with a data source like mechanomyography (MMG) and electromyography (EMG), which offer valuable insights into movement, yields successful outcomes through the application of artificial intelligence (AI). Machine learning (ML) and deep learning (DL) algorithms in artificial intelligence (AI) can identify hand movements. The aforementioned methods may necessitate differing quantities of data due to their structure. In this regard, machine learning can produce superior outcomes with limited data, whereas constructing a deep learning model necessitates extensive datasets [[1], [2], [3]].

Throughout recent decades, gesture recognition has emerged as a vital component of interaction between humans and computers (HCI), improving user engagement with technologies. The gestures chosen for our dataset were selected for their prevalent use and practical relevance across diverse fields. These gestures represent a varied spectrum of movements that might enhance intuitive interactions among smart home gadgets, game interfaces, and assistive technology. By supplying a thorough dataset that encapsulates these gestures, we intend to enhance research and development in gesture-based human-computer interaction, addressing current deficiencies in the existing literature.

Mechanomyography (MMG) signal datasets are crucial due to their unique characteristics and the techniques used for their acquisition. MMG is a non-invasive technique, with fewer sensors required, reduced sensor sensitivity, lightweight equipment, and a simpler setup. The signal-to-noise ratio improves, and the setup is straightforward and reliable. MMG uses mechanical vibration, eliminating the need for skin preparations, making skin impedance a non-problem. This sets MMG apart from other biosignal datasets like electromyography [4]. As most of these features of MMG signal acquisition techniques, some of which are related to the nature of the signal itself, can be considered important solutions to the problems that EMG signals and their techniques suffer from.

## 3 Data Description

Table 1 analysis, which includes datasets from the pertinent literature, shows that several factors and attributes were carefully considered when creating our proposed dataset, which enhances its robustness and applicability for various HCI applications. This dataset possesses a substantial number of subscribers, with investigations revealing a limited number of datasets that match or exceed this count. The subscribers span a diverse age range and exhibit a significant female representation, resulting in a relatively balanced demographic. Notably, a considerable portion of participants is left-handed, a characteristic not commonly observed in previous datasets. Furthermore, the dataset includes a mix of athletes and non-athletes, along with an extensive collection of gesture samples from each participant, culminating in a vast total of samples within the dataset. The collection of gesture examples for each participant was intentionally spread over numerous sessions on different days, rather than being completed in a single day or session. To augment realism, the samples from individuals were gathered in diverse, practical, and authentic settings, rather than in a meticulously curated optimal environment. The point is to make it clear that numerous research projects focused on dataset creation often omit critical details pertinent to the dataset, which researchers require for evaluation and application across various fields, as exemplified in Table 1. The lack of these details and information may be considered a limitation that could impede the acquisition of supplementary discoveries, potentially enhancing the research using these datasets [4].

**Table 1:** Literature's mechanomyography (MMG) based datasets.

ID	Ref.	a	b	c	d	e	f	g	h	i	j	k	l	Year
1	[5]	11	NS	NS	NS	NS	12	1320	13	Accel+gyro	NS	LE	HGR	2019
2	[6]	35	16-55	NS	NS	NS	8	14,000	1	Accel	NS	NS	HGR	2020
3	[7]	6	22-39	NS	NS	NS	7	252	2+2	Accel+microphone	NS	LE	HGR	2020
4	[8]	10	22-30	50 % -M 50 % -F	100 %-R	NS	14	1400	1+1+10	Accel+gyro+microphone	2-3	NS	HGR	2020
5	[9]	1	NS	NS	NS	NS	8	160	6+1	Accel+gyro	NS	LE	HGR	2021
6	[10]	14	25-53	57 % -M 43 % -F	100 %-R	57 % -A 43 %-nA	7	NS	1+1	Piezo-plate +strain gauge	NS	NS	FMR	2021
7	[11]	20	NS	NS	NS	NS	18	18,000	1+1	Accel+gyro	5	NS	HGR	2021
8	[12]	4	NS	75 % -M 25 % -F	NS	NS	30	6000	16+16+16	Accel+gyro+magnometer	NS	NS	HGR	2023
9	[13]	NS	NS	NS	NS	NS	15	5204	5+5	Accel+flex sensor	NS	NS	SLR	2024
10	[14]	8	22-29	100 %-M	NS	NS	4	4800	8	Accel	12	NS	HGR	2024
*		43	18-69	60 % -M 40 % -F	79 % -R 21 % -L	65 % -A 35 %-nA	11	23,650	1+1	Accel+gyro	3-4	LE EO HA Gym	HGR	2025

* - this dataset.

a: the number of participants.

b: age range.

c: the gender distribution (Male (M) / Female (F)).

d: the dominant hand (Right (R) / Left (L)).

e: the physical activity (Athletic (A) / non-Athletic (nA)).

f: the number of adopted gestures.

g: the total count of gesture examples executed to compile the dataset.

h: the number of sensors used to capture data.

i: types of sensors.

j: the number of days or sessions required to collect data from each participant.

k: the environment in which the participants were sampled.

l: The application utilizing the dataset.

NS: Not-Stated.

LE: Laboratory Environment.

HA: Home Ambiance.

EO: Employee Office.

HGR: Hand Gesture Recognition.

SLR: Sign Language Recognition.

FMR: Finger Motion Recognition.

**Fig. 1:**
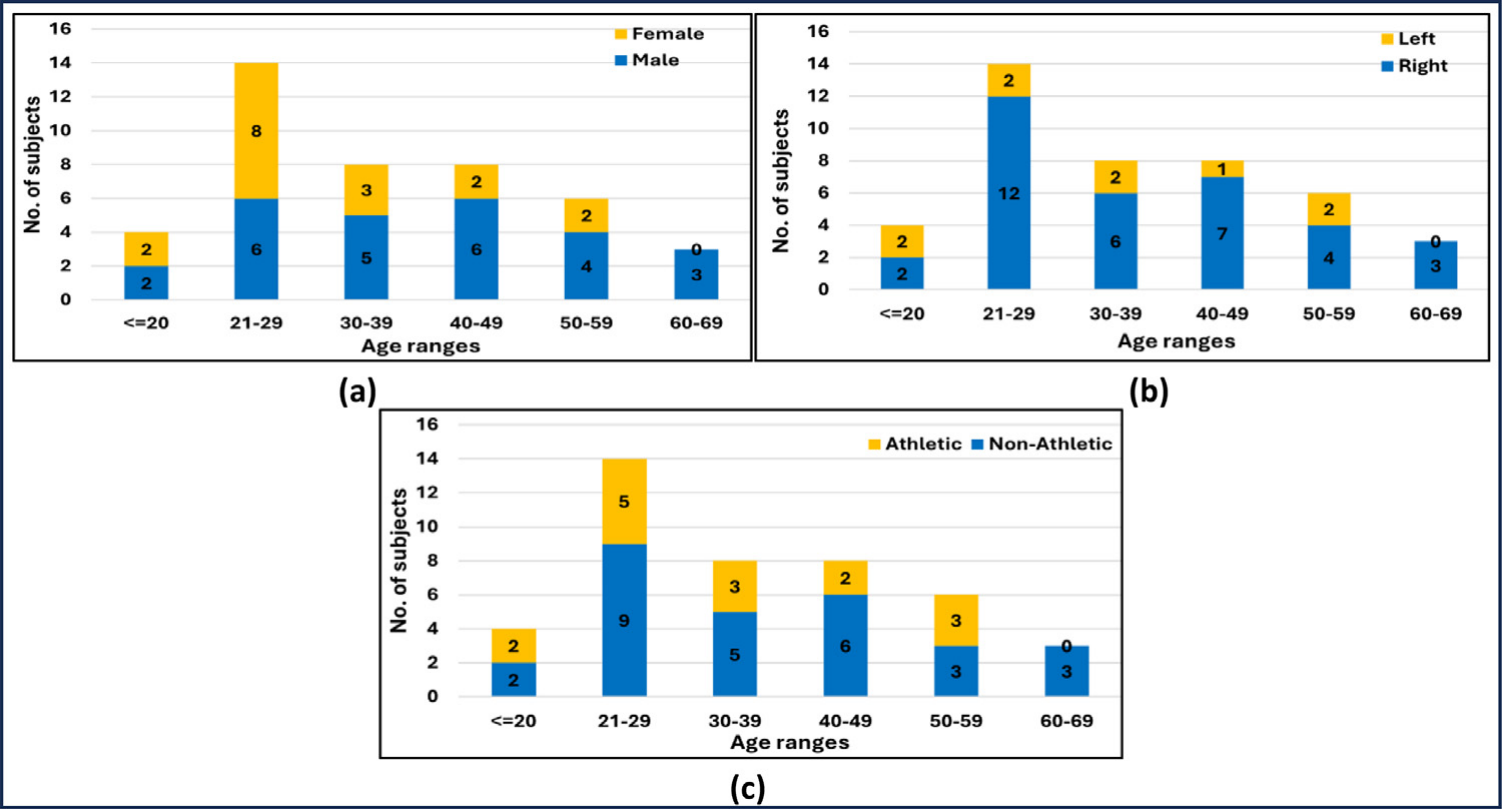
Demographic distribution of participants by: (a) age and gender, (b) age and dominant hand, (c) age and physical activity.

This article presents a dataset comprising data from 43 healthy volunteer participants (26 men and 17 women), reflecting a distribution of 60 % to 40 %, respectively. The participants span a broad age range of 18 to 69 years and are categorized by dominant hand (34 right-handed and 9 left-handed), yielding a ratio of 79 % to 21 %. Additionally, they are classified based on physical activity levels (28 non-athletic and 15 athletic), resulting in a distribution of 65 % to 35 %. Fig. 1 illustrates the demographic distribution of the participants, organized by age groups in relation to gender, dominant hand, and physical activity status.

**Fig. 2:**
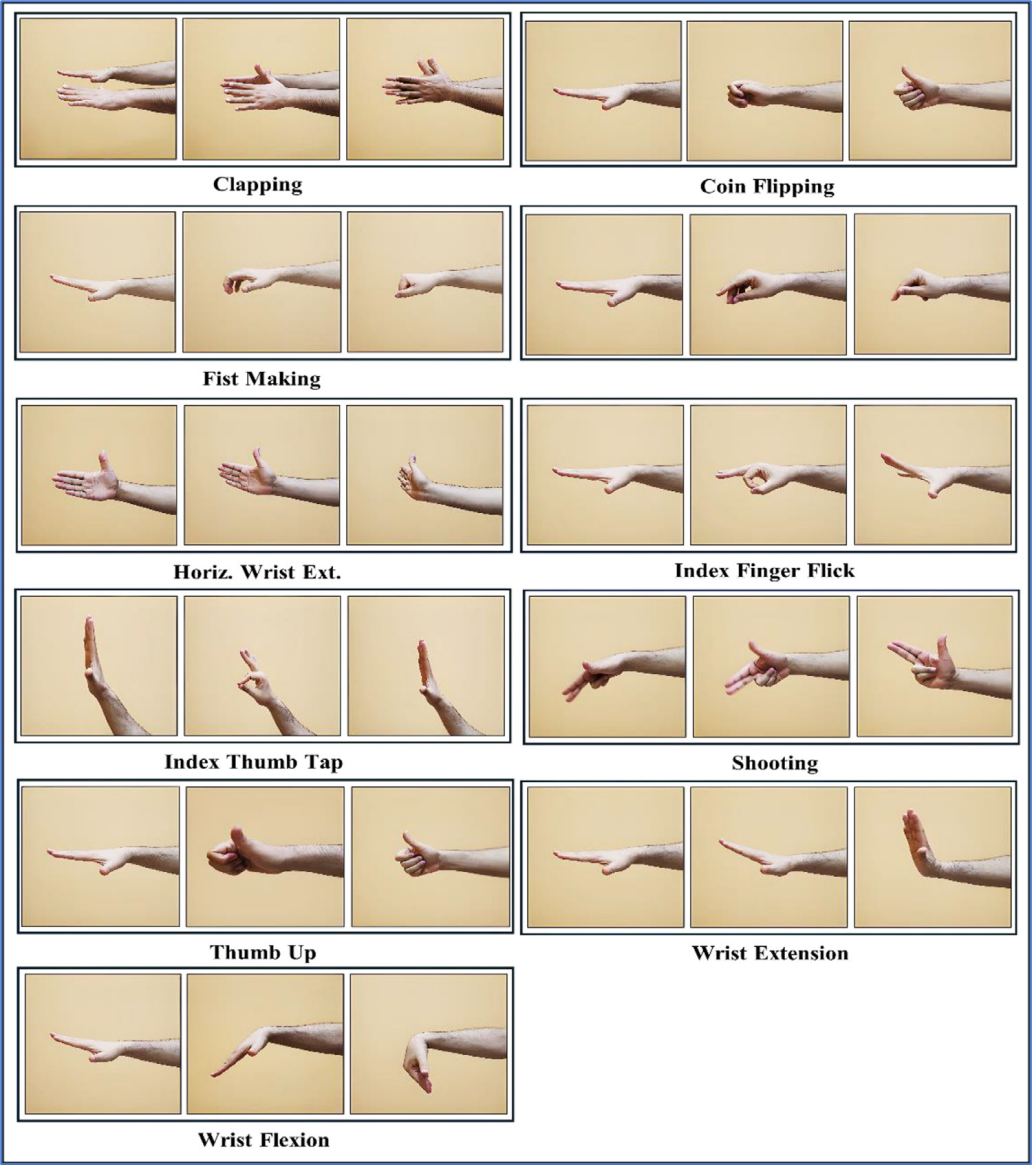
The implementation of the 11 proposed gestures.

All participants were prepared, trained, and instructed to execute 11 significant gestures applicable in many contexts and strongly associated with daily life necessities. The gestures include clapping, coin flipping, finger snapping, fist making, horizontal wrist extension, index finger flicking, index thumb tapping, shooting, thumbs up, wrist extension, and wrist flexion, as illustrated in Fig. 2, which depicts the execution of each gesture through multiple images.

A wide variety of motions, each with its own special place in the history of HCI, are part of our dataset. Bringing both hands together in a clapping motion is a typical way to gain someone's attention. Gameplay decision-making situations can benefit from the rotating motion of the wrist, which is required for coin flipping. In interactive systems, the finger-snapping gesture—which is made up of rapidly pushing the thumb and middle finger—is a useful trigger. A firm signal for positive acts is the formation of a fist, and for accurate control in many contexts, the horizontal extension and flexion of the wrists are essential. The use of flicking and tapping motions with the index finger improves interactions with touch-based interfaces. In gaming, the shooting motion is helpful because it mimics pulling a trigger, but in social interactions using technology, the thumbs-up gesture is beneficial because it indicates approval. The use of high-quality sensors to record each movement guarantees accurate information for algorithms that recognize gestures.

The data capture process is designed to be as realistic as possible, reflecting the actual performance of each participant in an environment that is not ideal and is not prepared in advance. Rather, the signals are collected while the participant is conducting their daily activities in the location of their choice. The data of each participant was collected in three to four sessions on separate days, with varying arrangements. For instance, one participant may perform all of the gestures with all of their repetitions on three or four consecutive days, while another participant may perform a session every week or every three days. Consequently, the gestures and their performance times were divided according to a pre-determined schedule for each participant.

The collection of hand gesture data from several places introduces considerable environmental heterogeneity that significantly affects signal quality and, consequently, classification performance. Acquiring signals in varied environments—such as residences, fitness centers, and outdoor locations—subjects biological signals (e.g., MMG, EMG, and EEG) to variations in illumination, temperature, ambient noise, and humidity. These conditions can compromise signal integrity, leading to elevated noise levels and fluctuating amplitude characteristics. For instance, elevated ambient noise levels may add distortions into the data, while fluctuations in humidity might affect sensor performance, among other factors. In a regulated laboratory environment, signals are generally more pristine and uniform. Conversely, signals recorded in domestic or gym settings may demonstrate more variability because of interference from external sources (e.g., motion, background discussions). The context of gesture performance may differ, resulting in variations in execution style; for instance, gestures in a gym may be more vigorous than those in a tranquil home. Various contexts may affect the execution of gestures. A volunteer may execute a gesture differently in a gym (more expansive environment, potentially greater energy) compared to at home (more restricted space, likely more relaxed). Because things are hard to predict, classification models need to be trained on a wide range of datasets to make them more reliable and useful in real life. By amalgamating data from diverse locations, researchers can construct models that are more adept at recognizing gestures across various contexts, hence enhancing accuracy and applicability in real-world situations characterized by unpredictability. This all-encompassing method helps us fully understand how environmental factors affect physiological responses, which leads to better and more adaptable systems for recognizing gestures.

In accordance with the aforementioned concepts, the data for this dataset was collected in four primary locations: the participant's residence or workplace, the gym, or one of the university laboratories. This pertains to certain participants, including instructors and personnel in the College of Engineering's departments.

**Fig. 3:**
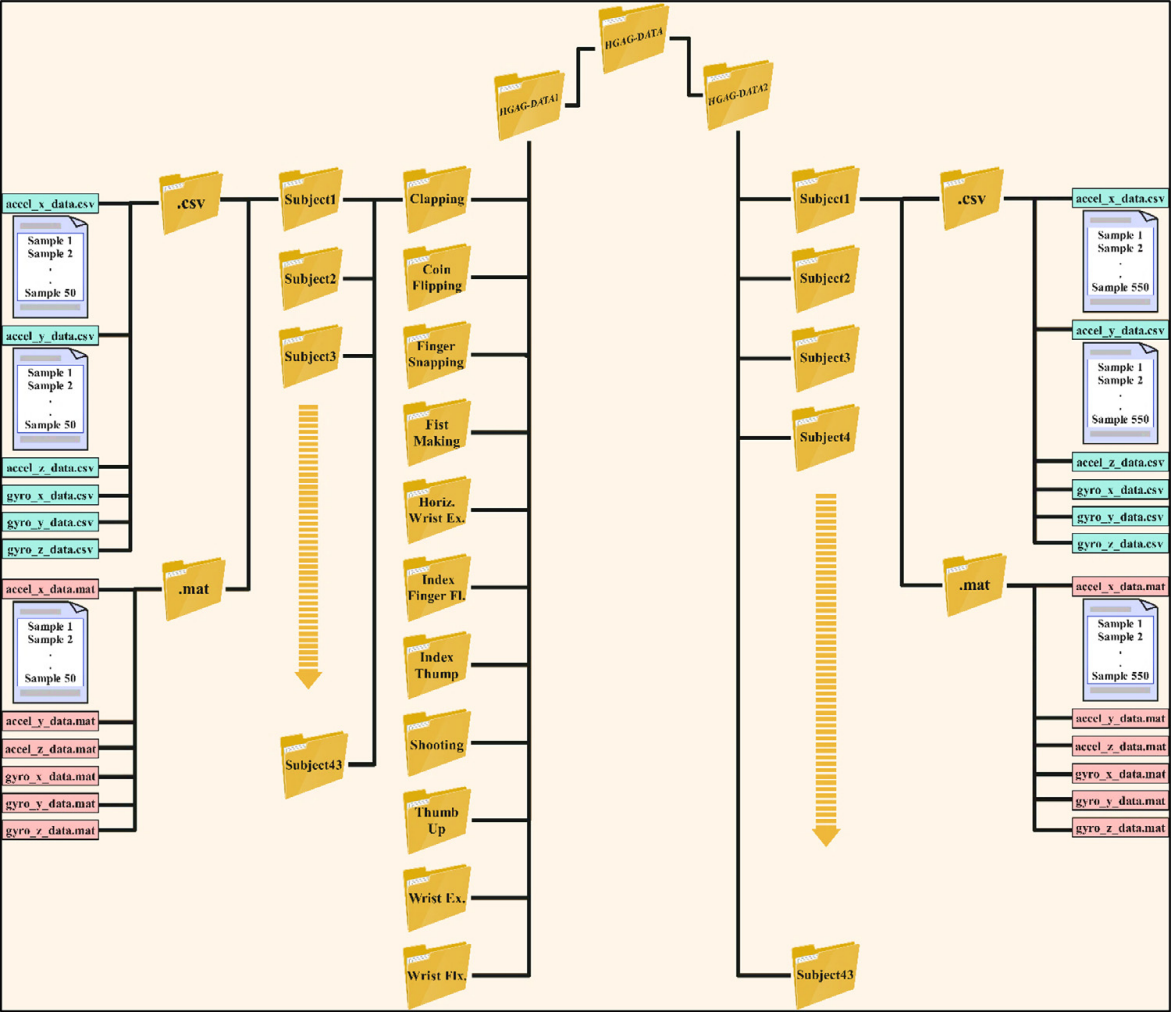
Schematic diagram of the HGAG-DATA dataset.

The data was organized into two hierarchical structures within the main folder labeled (HGAG-DATA), corresponding to the dataset's name, to facilitate access, collection, and manipulation. Fig. 3 demonstrates how the first method separated the data based on gesture type, while the second method separated the data based on participant number. The researcher can access data for each gesture and all participants by navigating to the folder (HGAG-DATA1). This folder contains eleven subfolders, each corresponding to one of the eleven gestures proposed in this study, along with 43 secondary folders representing individual participants (Subject_1, Subject_2, …, Subject_43). Each folder contains two directories: one named csv, which holds accelerometer and gyroscope data in CSV format, and another named mat, which contains the same data in MAT format. Each set comprises six data files with ``.csv'' and ``.mat'' extensions, respectively. The six files for each of the two formats contain data for the x, y, and z axes of both the accelerometer and gyroscope named as follows: accel_x_data, accel_y_data, accel_z_data, gyro_x_data, gyro_y_data, and gyro_z_data. Each file includes 50 repetitions of a specific gesture along one of the six axes, as captured by the accelerometer and the gyroscope. Consequently, there are six samples for each repetition distributed across the six files.

**Fig. 4:**
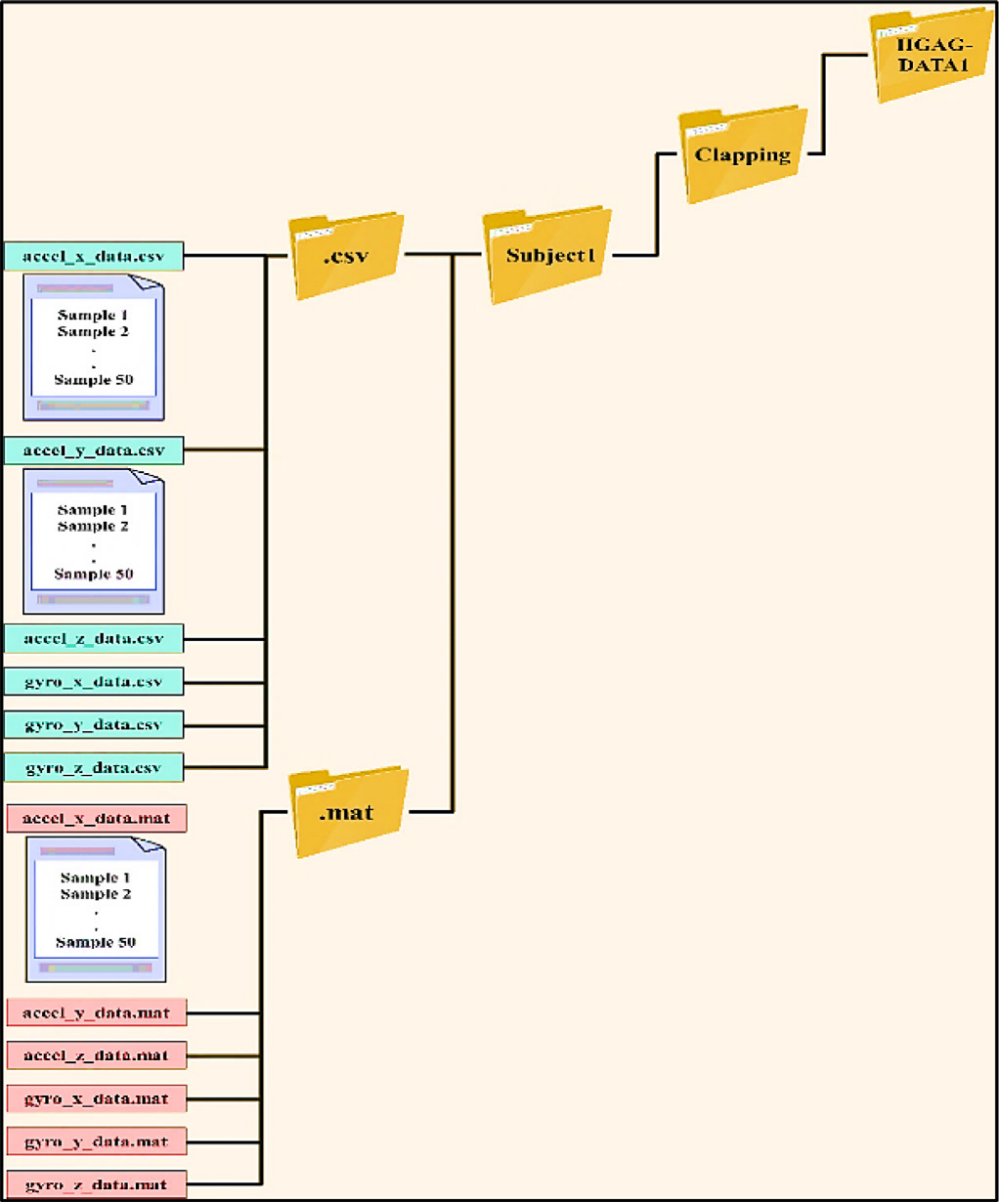
Schematic diagram of the clapping gesture.

For instance, Fig. 4 more closely depicts the data collected for subject1 following fifty clapping gesture repetitions; his data is split across six files with the CSV extension and six additional files with the same content but with the MAT extension. This process is repeated for all the other participants and all the remaining gestures.

**Fig. 5:**
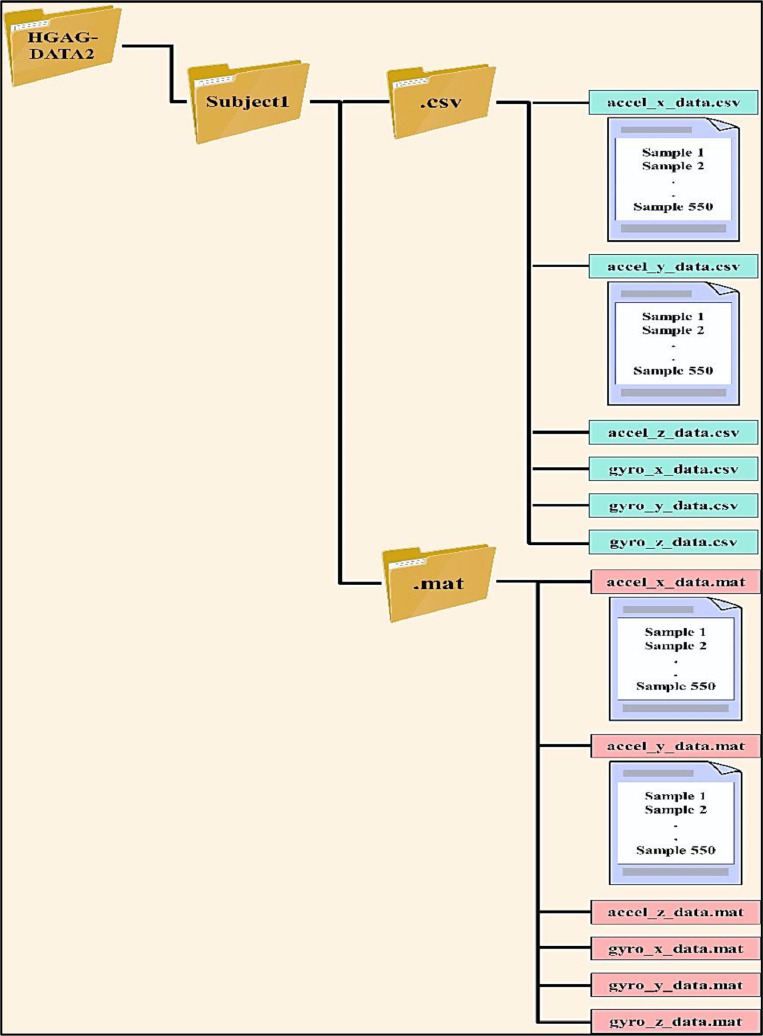
Schematic diagram of subject1.

The data in the second hierarchical structure is organized by participant names in a folder named HGAG-DATA2. There are forty-three subfolders in this folder, one for each participant. Two secondary folders, csv and mat, each containing six files representing data from three axes of the accelerometer and three axes of the gyroscope, are contained in each subfolder. Every file contains information on all eleven motions, each of which is performed fifty times, for a total of 550 samples (11×50) for every participant. Structuring the data in each file to depict 50 successive repetitions of each gesture in the subsequent order: clapping, coin flipping, finger snapping, fist formation, horizontal wrist extension, index finger flicking, index thumb tapping, shooting, thumbs up, wrist extension, and wrist flexion. As a result, all volunteer data is gathered in a different folder by the second organizational structure. Fig. 5 illustrates the arrangement of participant 1′s data within a singular folder, encompassing recorded samples of this individual executing all gestures and their repeats. The information of all the remaining participants is stored uniformly.

The primary directory (HGAD-DATA) now comprises two subdirectories housing the complete dataset (HGAG-DATA1 and HGAG-DATA2), a folder titled ``Hand gestures images'' holding images of the execution of each of the 11 gestures utilized in this study, and four additional files. The initial file, questionnaire.docx, contains a replica of the inquiries shown to all participants in a distinct session before commencing the data collection processes. The objective of this method was to identify persons suitable for dataset development and to collect requisite demographic information from the participants. This first questionnaire effectively eliminated two individuals who consented to perform and record motions. These individuals exhibited neurological disorders, characterized by a pronounced tremor in their hands during the execution of motions, which substantially hindered their capacity to perform these movements consistently. Furthermore, one person who experienced hand nerve discomfort was excluded. The pain radiates through the wrist when executing movements. The second file, titled ``Questionnaire results.xlsx,'' includes the responses of all selected participants to the specified questionnaire questions. The third file contains the consent form template that was distributed to all participants, each of whom signed a copy prior to the commencement of the data recording cycles. The fourth file, titled ``Schematic Diagram of the HGAG-DATA Dataset.docx,'' includes illustrations and a comprehensive description of the dataset's organization and the architecture for data storage.

## 4 Experimental Design, Materials and Methods

### 4.1 Preparatory stage

To select suitable participants for this study, each volunteer was individually met in a separate session prior to the recording of the adopted gestures, lasting between 30 and 45 minutes. During this time, responses from the nominated participant were collected for nine distinct questions of the “questionnaire.docx” file, as detailed in Table 2. Several of these inquiries pertain to his biological attributes, including his name, age, gender, the dominant hand utilized in daily activities, wrist circumference, and overall health status, encompassing his physical activity level, athletic involvement, and any medical conditions that may hinder his full participation. The participant is inquired about any diseases or symptoms affecting the muscles and nerves throughout his body, particularly in the hands, and whether such conditions necessitate medication that may influence his ability to perform the required gestures repeatedly over multiple sessions. The outcomes of the responses have also been uploaded to the database main folder (HGAG-ADAT) included in the file named “Questionnaire results.xlsx” alongside a copy of the pertinent questions.

**Table 2:** A sequence of questions to ask before acquiring signals.

Question#	Statement of the question	Potential answers
1	What's your name?	String value
2	How old are you?	Numerical value
3	What's your gender?	• Male (M) • Female (F)
4	What's your wrist circumference?	Numerical value in centimeters
5	Are you an athletic person?	• Yes • No
6	Which hand do you predominantly use?	• Right (R) • Left (L)
7	Do you have any medical conditions or health issues that hinder your ability to participate in this type of study?	• Yes • No
8	Do you suffer from any muscle or nerve disease?	• Yes • No
9	Do you take any medications or drugs that affect muscle or nerve performance?	• Yes • No

The actual names of participants have been omitted from the table to maintain privacy, and have been substituted with the designation (Subject_n), where n denotes the participant number, ranging from 1 to 43. The study's purpose is articulated to the participants, specifying the total number, types, names, and repetitions of the gestures to be executed, along with an initial exercise where the volunteer replicates several repetitions of these gestures without wearing any devices after observing them demonstrated by the supervisor. The participant is succinctly introduced to the system comprising a series of devices that will be worn, along with the methodology for their interconnection and linkage to the computer utilized for displaying and storing the captured signals. Subsequently, the volunteer dons the devices on the dominant arm and executes the requisite gestures through simple repetitions, while receiving feedback regarding the accuracy of his performance and instructions for rectifying any movement discrepancies.

Upon the participant's acclimatization to all work components, an assessment is obtained from each volunteer regarding the gestures that posed challenges during practical execution, as well as those that were straightforward. Additionally, the preferred sequence of gestures, including their repetitions, is noted, as variability among volunteers in this aspect has been observed. The gestures are organized into a schedule spanning three to four days, tailored to the abilities and preferences of each participant. This calendar outlines the session dates, including date, time, and the quantity of gestures executed together with their repetitions in each session, ensuring a degree of equilibrium in the number of gestures performed each time.

### 4.2 Equipment preparation stage

The extensor and flexor muscles of the arm are essential for executing diverse movements. Given that these sets of muscles work together to allow for a broad range of expressive and practical gestures in everyday engagement and communication. Muscle contractions produce mechanical vibrations resulting from the oscillations of muscle fibers and adjacent tissues. The mechanical signals produced by the contraction of these muscles are referred to as mechanomyography signals (MMG). MMG offers a comprehensive overview of mechanical reactions, which can enhance the understanding of muscle and movement dynamics. This may encompass data from specialized sensors that directly measure muscle activity, such as piezoelectric sensor, microphone, laser distance sensor, and accelerometer. The latter has demonstrated efficacy in numerous research concerning applications linked to hand gestures and their identification, including touchless interfaces, gesture-based controls in gaming and prosthetic devices, as well as smart technology. This is attributable to its numerous advantages, the foremost being its provision of a non-invasive method of measuring muscle activity, rendering it appropriate for wearable applications. It is typically more cost-effective and readily accessible than specialized MMG sensors, and can be seamlessly integrated into other sensors (like gyroscopes) to deliver a holistic perspective on muscle activity and overall movement patterns. This synergistic method facilitates the acquisition of both mechanical vibrations from contractions of muscles (MMG component) and motion dynamics (kinematic component). Consequently, integrating them can yield a more thorough comprehension of gesture performance while improving the accuracy, usefulness, and applicability of gesture recognition systems, resulting in more effective and inventive solutions. In hand gesture recognition, an MMG (mechanometry) dataset often denotes data acquired from muscular contractions, frequently with sensors positioned on the skin above the muscles.

**Fig. 6:**
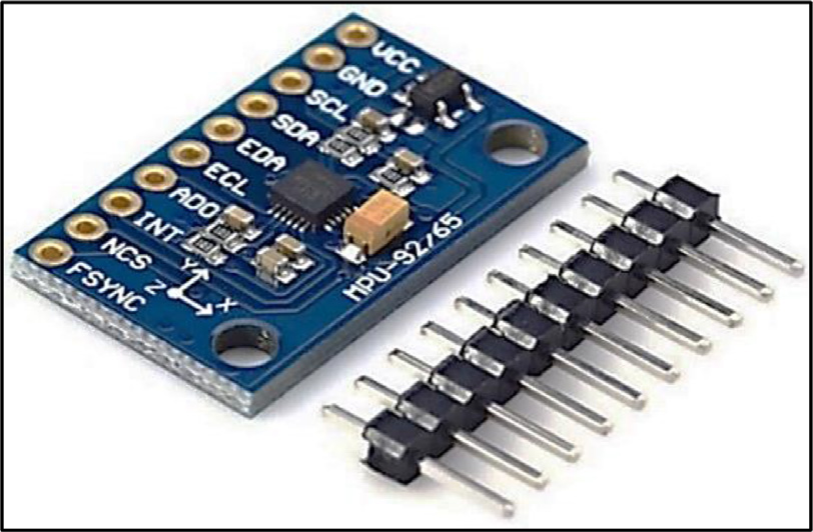
MEMS MPU-92/65 sensors module.

In this study, the data of the ``HGAG-DATA'' dataset has been captured by using a novel human-computer interaction (HCI) system that employs the tri-accelerometer and tri-gyroscope sensors of the MPU- 92/65 sensors module, as shown in Fig. 6. The MEMS MPU-92/65 sensors module is categorized as a motion tracking device, developed by InvenSense Inc. (now part of TDK Corporation). The System in a Package (SIP) includes a 3-axis accelerometer, gyroscope, magnometer, and an integrated digital motion processor (DMP) capable of handling complex sensor fusion algorithms. Due to its small form factor, high degree of accuracy, and low power consumption, the MPU-92/65 is widely used in a variety of applications, such as health, fitness, and athletic wearable sensors, robotics, drones, mobile and handheld video games. It supports nine degrees of freedom. This chip communicates using the I2C (inter-integrated circuit) protocol [15,16]. Table 3 details the specs of the accelerometer and gyroscope sensors included in the MPU-92/65 module.

**Table 3:** Specs of the accelerometer and gyroscope.

Power voltage	Accel operating current	Accel range	Accel sensitivity	Gyro operating current	Gyro range	Gyro sensitivity	Sleep mode current	Integrated ADCs	Size	Weight
3 ∼ 5V	450µA	Up to ±16g	Up to 16,384 LSB/g	3.2mA	Up to ±2000 deg/sec	Up to 131 LSB/deg/sec	8µA	16-bit	15×25mm	2.72g

**Fig. 7:**
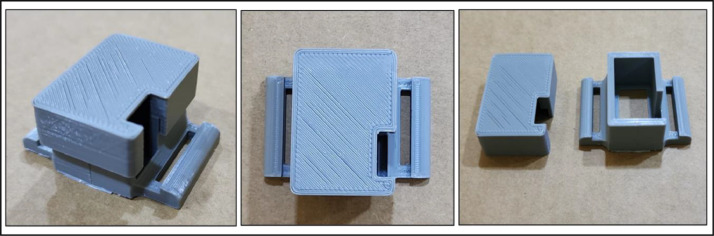
3D-printed container for the MPU-92/65 module.

The MPU-92/65 module, responsible for sensing and measuring signals, has been designed in the form of a standard wristwatch. This design allows each participant to wear it conveniently, standardizing the wearing process and eliminating issues related to sensor placement and misalignment. The MPU-92/65 is securely housed in a compact case resembling a wristwatch (L:31mm, W:21mm, H:15mm, Thickness:2.5mm). This case was designed with the 3D AutoCAD program, version 2022, and subsequently produced using a MALYAN MA10 mini 3D printer, as illustrated in Fig. 7.

**Fig. 8:**
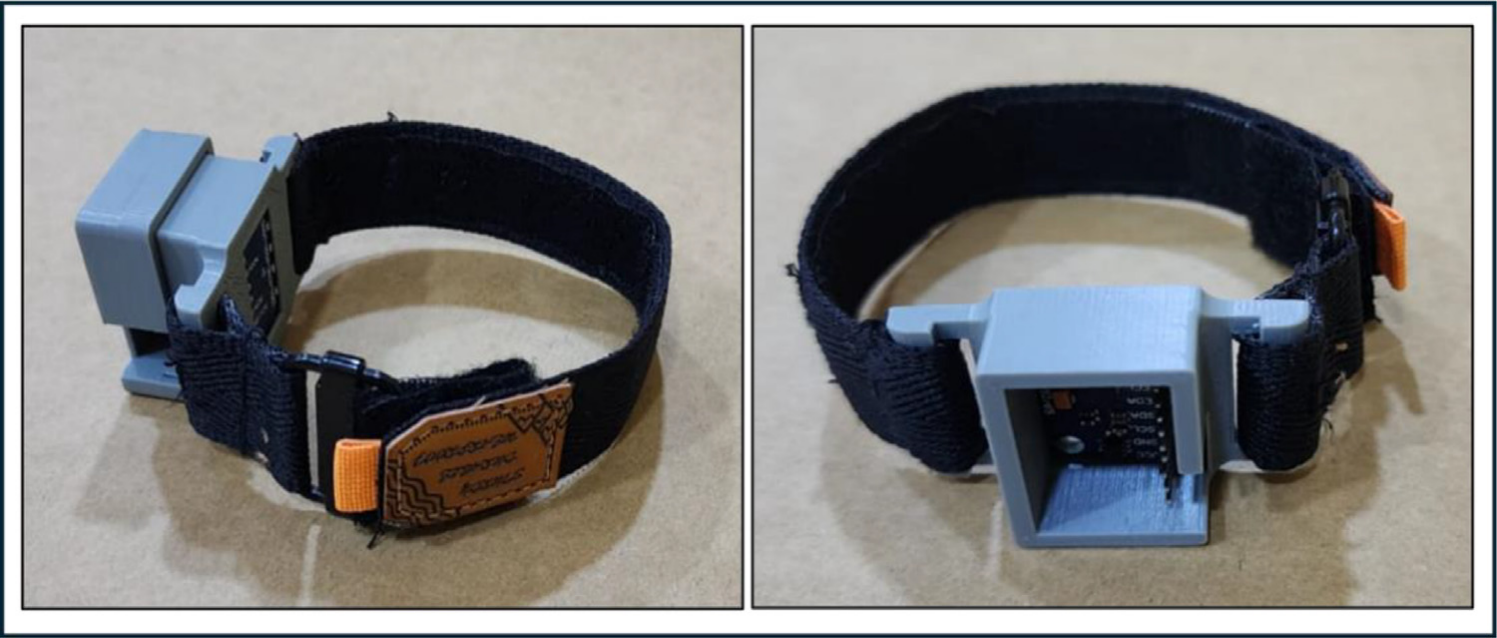
Final configuration of the MPU-92/65 wristwatch

A ``Watch Band'' brand watch strap has been installed in this case. This sports watch strap is constructed from a soft, durable, and environmentally friendly elastic material suitable for human skin. Fig. 8 illustrates the final configuration of this segment of the HCI system, featuring the MPU-92/65 sensor module integrated within it. The case is positioned atop the posterior ulna, which features a flat surface, ensuring seamless contact between the watch case and the skin. Consequently, the signals from multiple subjects can be measured with robustness and consistency. This location resembles the conventional watch position, facilitating ease of wear and ensuring uniformity in signal strength captured from various subjects.

Four female-to-female jumper wires connected the MPU-92/65 module to the first ESP32 DEVKIT V1 microcontroller (transmitter), which powers the sensor module and acquires data from it at sampling frequency 200Hz. This data, comprising accelerometer and gyroscope readings across three axes, is transmitted to the second ESP32 DEVKIT V1 microcontroller (receiver) via a Wi-Fi connection, which is directly linked to the laptop through a USB cable. The transmitter and receiver microcontrollers were programmed using Arduino IDE version 2.3.2 following the installation of the necessary libraries. The ESP32 is a series of microcontroller chips developed by Espressif Systems (Shanghai) Co., Ltd. built with the “Xtensa LX Microprocessor” [17]. Table 4 illustrates the specifications of the ESP32 DEVKIT V1 microcontroller.

**Table 4:** Specs of the ESP32 DEVKIT V1 microcontroller.

No. of cores	Wi-Fi	Bluetooth	Architecture	Clock freq.	RAM	Pins	Peripherals
2 (dual core)	2.4GHz up to 150 Mbits/s	BLE and legacy Bluetooth	32 bits	Up to 240MHz	512KB	30	ADC,DAC,I2C,UART,CAN, 2.0,SPI,I2S,RMII,PWM

MMG identifies and analyzes the mechanical activity (vibration) of contracting muscles. Active skeletal muscle fibers transmit information through low-frequency lateral oscillations. Researchers have demonstrated that the signal's normal frequency range is 3 Hz to 100 Hz [4,18]. The majority of earlier research employed sensors with sampling frequencies of 1000 or 2000 Hz. This study uses a 200 Hz sample frequency to lower the power consumption and computing load of the signal processing.

**Fig. 9:**
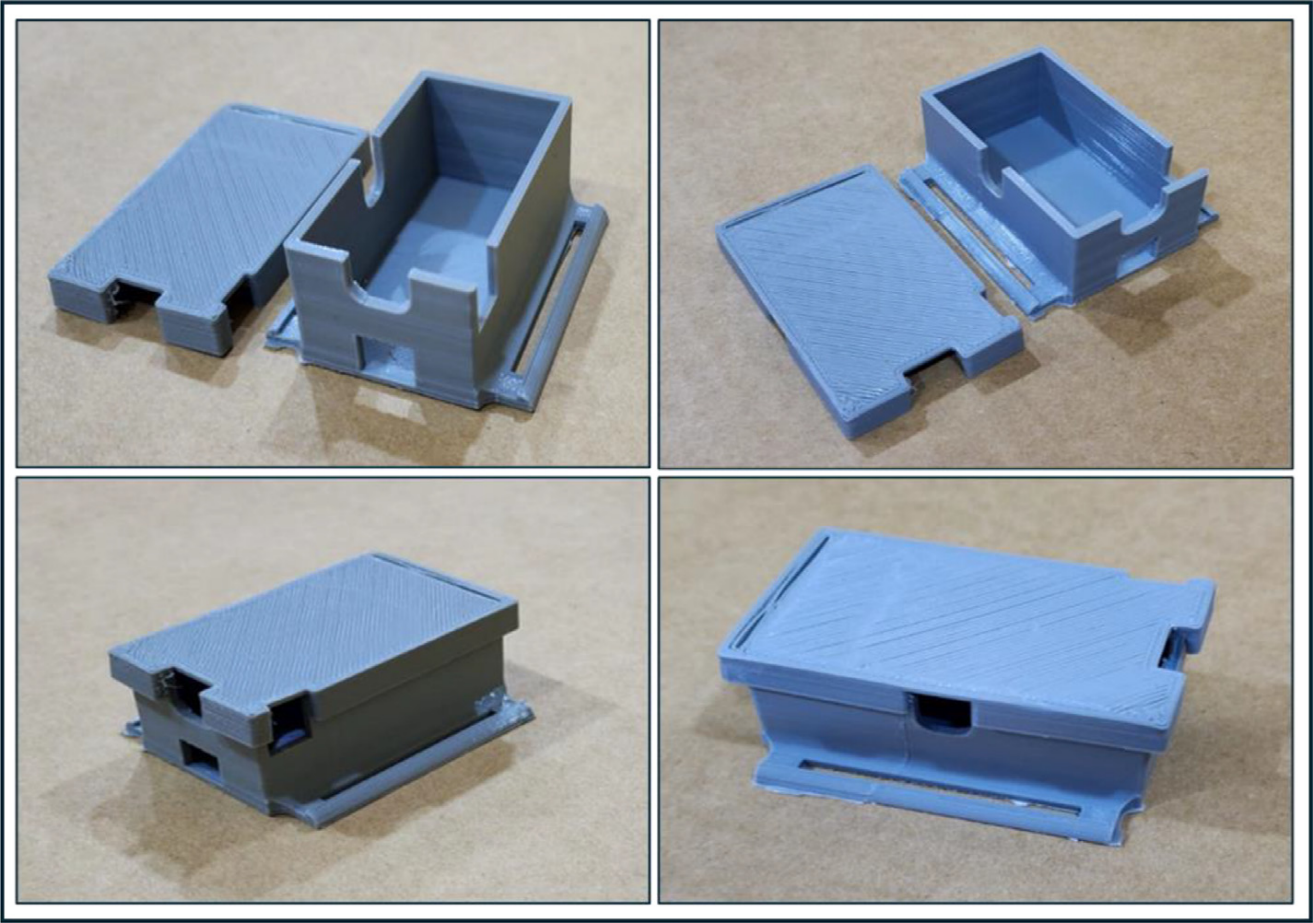
3D-printed container for the transmitter, battery, and battery charger.

**Fig. 10:**
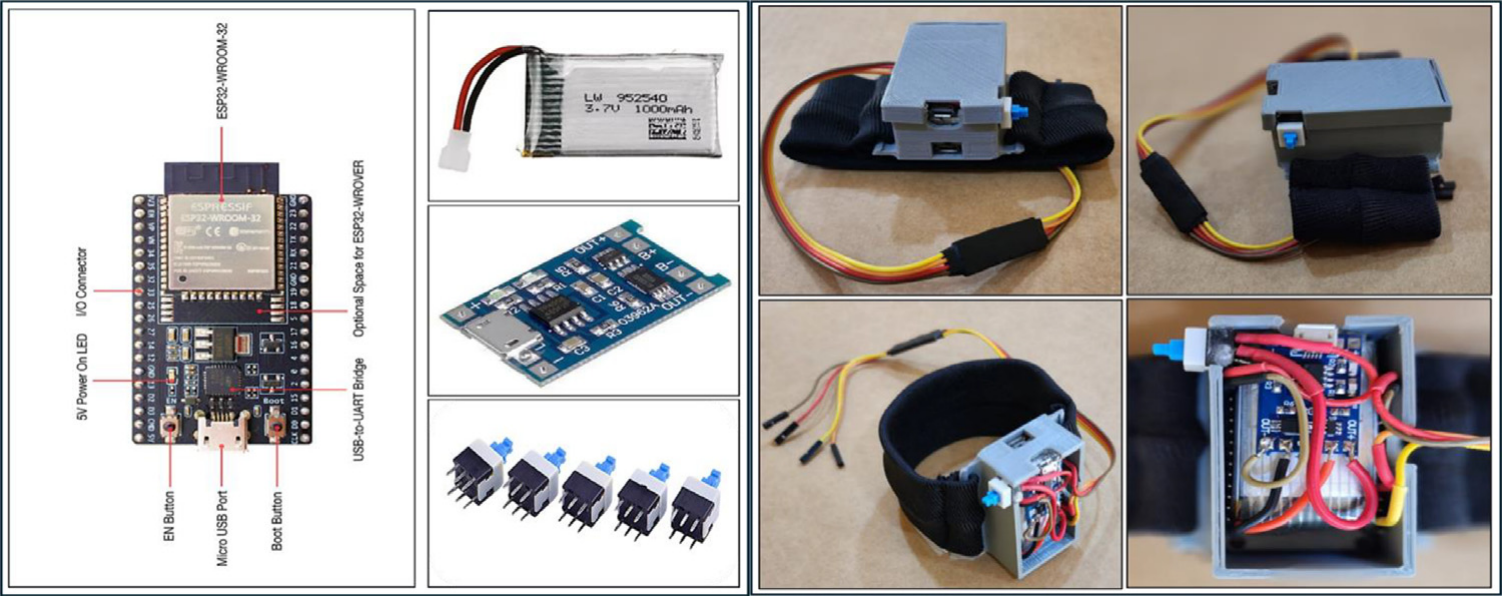
Final configuration of transmitter’s block.

To ensure a certain level of comfort, autonomy, and freedom of movement, as well as to avoid being directly connected to the laptop while executing gestures with their numerous repetitions. The AutoCAD application and a 3D printer were employed to create a small and compact 3D-printed case (L:56.5mm, W:33mm, H:22mm, Thickness: 2.5mm). A durable strap, which was sewn onto the box, is designed to ensure that the volunteer can wear the box effortlessly and securely. The strap is composed of a combination of rayon, nylon, and cloth. In the case, the ESP32 microcontroller (transmitter), a small rechargeable battery, battery charger, and a push button self-locking switch (8×8×12mm) have been installed and secured, as illustrated in Fig. 9, Fig. 10. Table 5 presents the parameters of the 3.7V, 1000mAh lithium polymer (Li-Po) rechargeable battery marketed under the ``RC. BURST'' brand. Table 6 presents the specifications of the Micro–USB TP4056 lithium battery charger, a module charging board featuring dual protection functions.

**Table 5:** Specs of the lithium polymer (Li-Po) rechargeable battery.

Type	Battery capacity	Charging voltage	Working voltage	Weight	Dimensions	Working temp.	Rechargeable
Lithium polymer	1000mA	3.7 ∼ 4.2V	3.7V	22g	43×25×10mm	-10 ∼ 50C	Yes

**Table 6:** Specs of the battery charger.

Input voltage	Full charge voltage	Current	Charge precision	Input interface	Working temp.	Dimensions	Inversed polarity	LED indicator
4.5 ∼ 5.5V	4.2V	1A adjustable	1.5 %	Micro USB	-10 ∼ 85	25×19×10mm	No	R: charging G: full charged

**Fig. 11:**
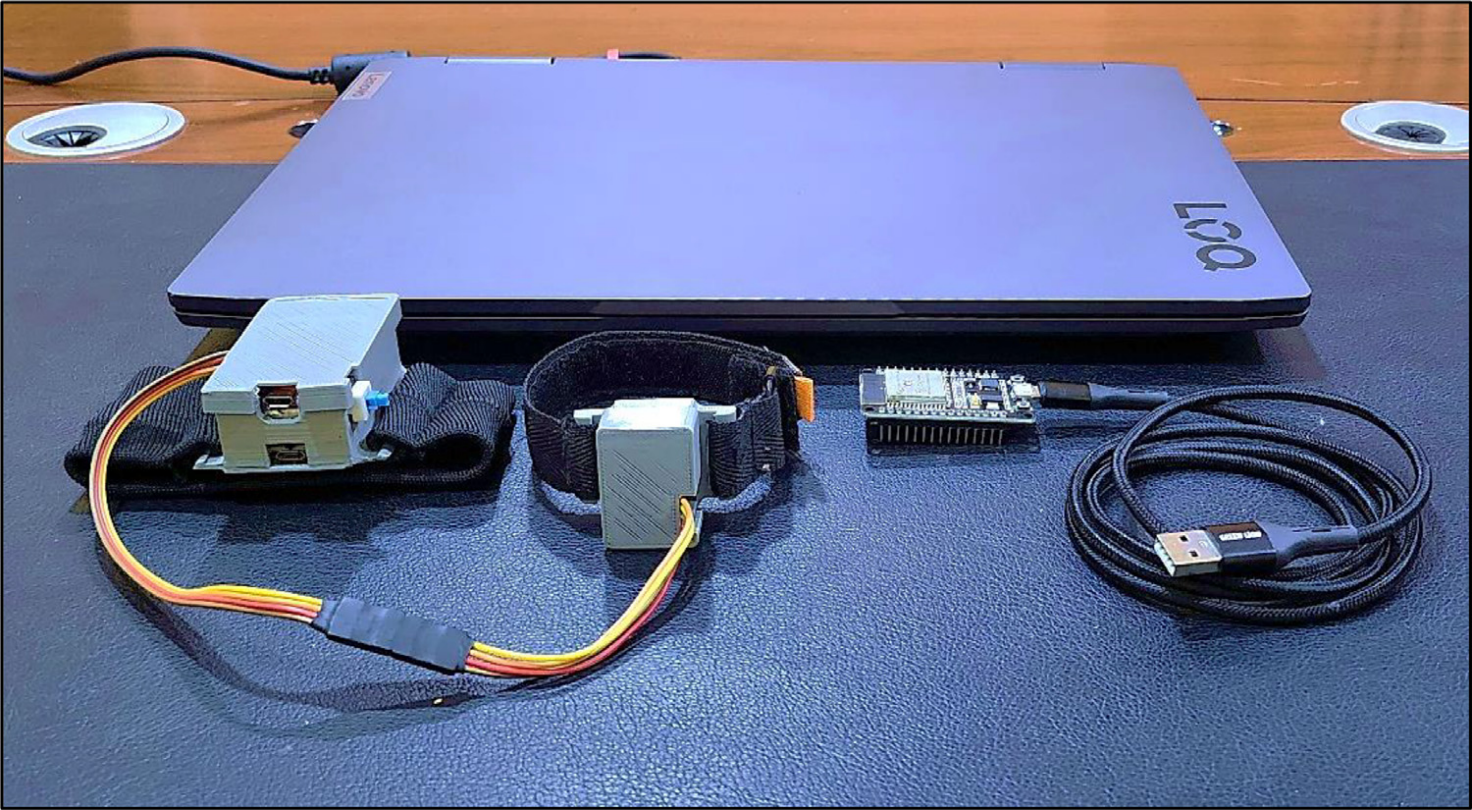
Hardware configuration of the system.

This dataset's human-computer interaction (HCI) system prototype comprises four primary components. The participants wear the first two components, a wristwatch strap with an MPU-92/65 sensors module and the first ESP32 microcontroller (transmitter), on their dominant hand during movement execution. When the volunteer performs a specific gesture, both the accelerometer and gyroscope detect signals along three axes and transmit them via I2C pins to the transmitter, which captures the signals at a sampling frequency of 200 Hz. Subsequently, the transmitter relays the signals to the second ESP32 microcontroller (receiver) through a Wi-Fi connection, which is directly linked to the laptop. The receiver and the laptop are the other two principal components of the system, as seen in Fig. 11, which depicts the system's hardware configuration.

Table 7 delineates the specs for the laptop required to analyze and store the data. The material prices for all components of the system, including sensors, microcontrollers, batteries, chargers, and wires, vary from 20 to 22 dollars, which is relatively inexpensive given the functionalities provided.

**Table 7:** Specs of the laptop.

Device type	Processor	RAM	System type	Operating system	GPU
Lenovo LOQ 15IRH8	13^th^ Gen Intel(R) Core (TM) i7-13700H 2.40GHz	16GB SDRAM DDR5 5200MHz	64-bit OS, x64-based CPU	Windows 11 Home edition	NVIDIA GeForce RTX 4060 8GB DDR6 RAM

### 4.3 Data acquisition stage

**Fig. 12:**
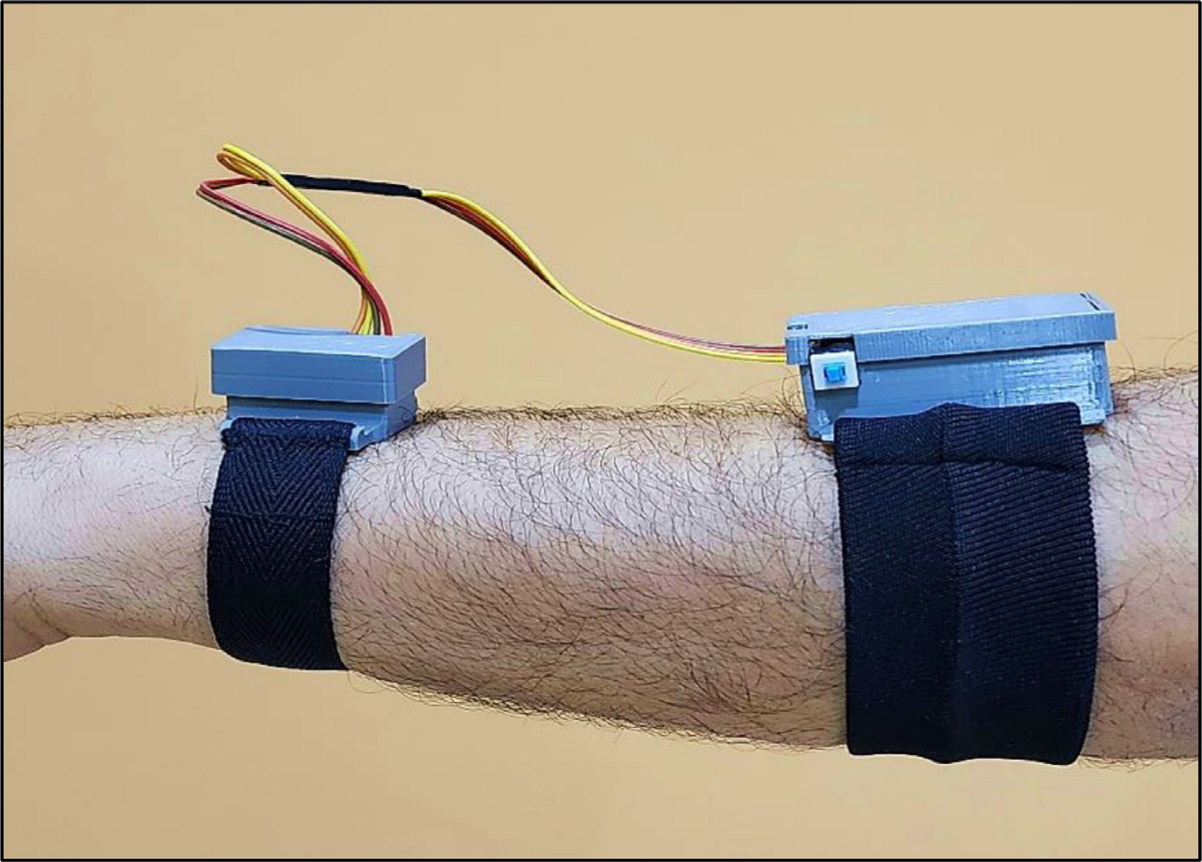
Components that participants wear.

The volunteer occupies a designated chair, positioned at an appropriate distance and oriented towards the table containing the laptop and the receiver microcontroller, which is directly connected via a USB cable. The volunteer dons the system components on their dominant hand, which consist of a printed case housing the transmitter microcontroller, a battery, and a battery charger. The volunteer positions the case on their forearm, maintaining a distance of 10 cm from the center of the elbow joint. The second part includes the watch strap that integrates the MPU-92/65 sensor unit. The volunteer positions it such that the center of the small case is 2 cm above the ulna, as shown in Fig. 12.

The volunteer positions her/his arm on the chair's armrest in a natural hand position, with the palm oriented downward. The session manager prompts participants with the names and repetitions of the gestures designated for that session, facilitating the execution of the necessary movements in his presence. Upon the volunteer's readiness to execute the gesture, the MATLAB R2023 script is initiated, facilitating the data retrieval from the receiver. This script is designed to ensure that each repetition of the gesture lasts for 1.25 seconds, during which 250 data points are recorded. Consequently, the volunteer is allotted one and a quarter second to complete a single repetition, a duration determined through experimental selection and extensive testing. Following numerous experiments, it was determined that the optimal timing for gesture execution occurs around the midpoint of the designated interval. Consequently, 0.3 seconds after the reading begins, the script activates a brief audio file that emits a beep sound, signalling the participant to commence the gesture.

The performance of 11 gestures, each repeated 50 times, was divided into four sessions for each volunteer to ensure data integrity and to simulate realistic gesture contexts while capturing their expressive signals accurately. During each session, every volunteer executed a segment of repetition for each gesture. During the initial two sessions, 13 repetitions of each gesture are executed, incorporating a 3-second rest interval between repetitions of the same gesture and a 2-minute break between different gestures, until all 11 gestures and their respective repetitions are finalized. During the third and fourth sessions, 12 repetitions of each gesture are executed, maintaining the same rest intervals between repetitions and gestures as observed in the first and second sessions. Each session is overseen by the individual tasked with receiving signals from the participants. Prior to the implementation of gestures, detailed instructions are provided, including reminders on their execution and sequence. Following each session, feedback is obtained from the volunteer concerning their performance, allowing for adjustments to the gesture sequences in future sessions based on the volunteer's preferences. This signal-capturing scenario also mitigates volunteer fatigue associated with performing numerous identical gestures concurrently, thereby enhancing performance consistency. If they executed all repeats of each gesture in isolation during a single session, they demonstrate strong performance in the early repetitions but subsequently encounter difficulties in the later repetitions, resulting in biased outcomes. This also diminishes the learning effects associated with repeated performances of the same gesture. Conversely, in this scenario, the volunteer is required to execute the same gesture across multiple sessions, resulting in a more natural performance. The same gesture is captured across multiple contexts, thereby improving the overall quality of the data. The implementation of this scenario necessitates significant mental preparation for frequent shifts in focus between gestures. Consequently, a limited number of volunteers experienced challenges in executing it correctly, failing to perform all gestures in each session. This led to the creation of a simplified scenario, eliminating the requirement for specific repetitions of all gestures in each session while striving to retain the benefits of the initial scenario as much as possible. In the second scenario, each gesture and its repetitions are executed in only two sessions. Each volunteer is assigned four sessions, during which the 11 gestures are distributed as follows: six gestures in the first and third sessions and five gestures in the second and fourth sessions. This enables the volunteer to concentrate on executing a limited number of gestures simultaneously. The volunteer executes 13 repetitions of the six gestures during the initial and third sessions, followed by a 10-minute rest interval. The six gestures and their repetitions are subsequently executed at the same pace. This principle is also applicable to the other five gestures in the second and fourth sessions.

**Fig. 13:**
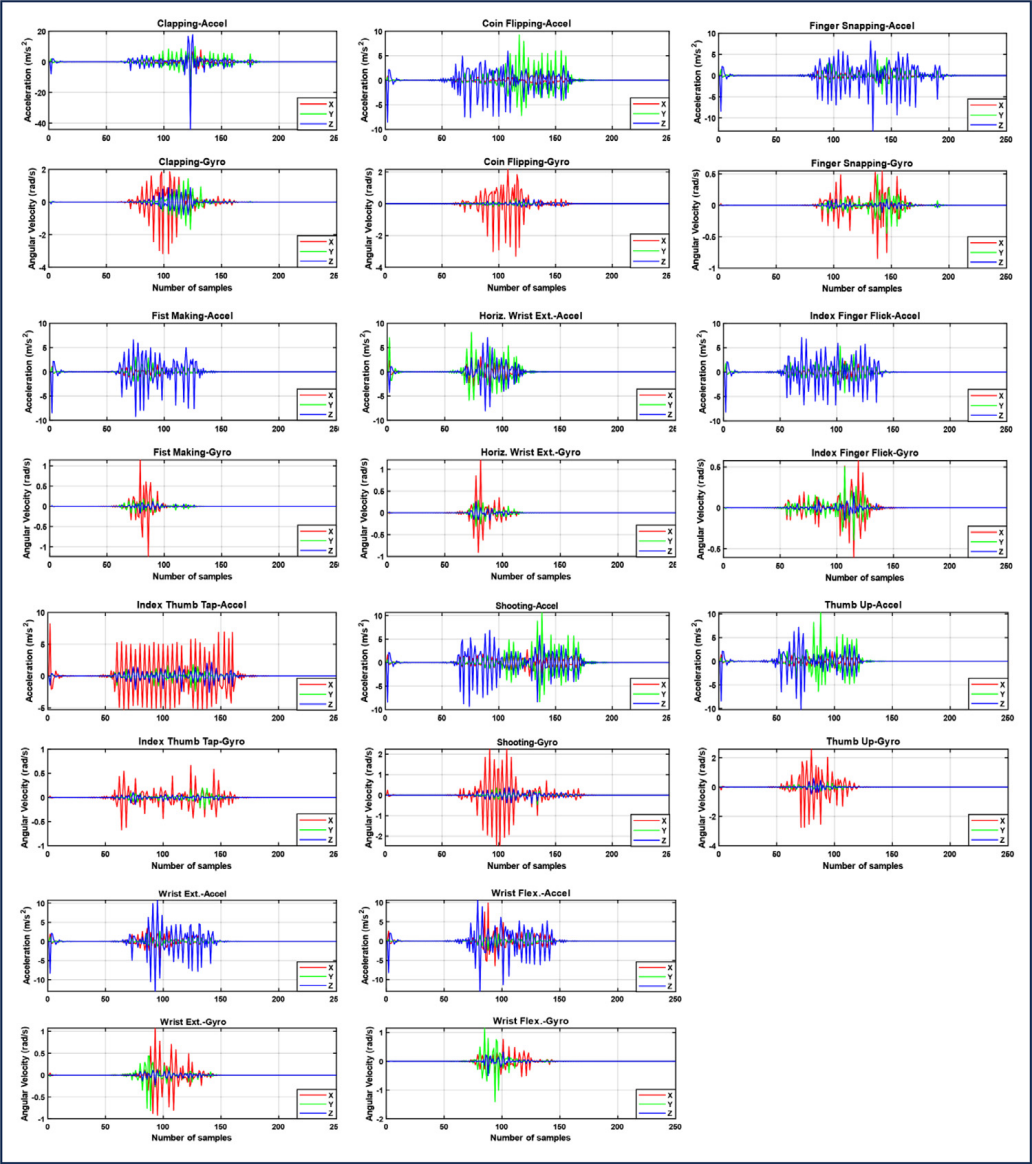
Acquired signals of the accelerometer and gyroscope of a participant during one repetition of each gesture.

The MATLAB R2023 script concurrently reads six signals, comprising three axes of accelerometer data and three axes of gyroscope data. A threshold value for the signal magnitude is used to make sure that the signal is recorded and stored consistently and of high quality, and to reduce the effects of bias on sensor readings. To ascertain the suitable threshold value without compromising the signals during actual gesture execution, numerous practical experiments were performed, resulting in the application of an absolute value of 0.5 m/s² for the accelerometer and 0.5 rad/s for the gyroscope. Consequently, any signal value falling inside the threshold range is recorded as zero. To get rid of unwanted motion artefacts and high-frequency noise while gestures were being executed, a 30-90 Hz 5th order Butterworth bandpass filter was applied to the raw data. The cutoff frequencies were set empirically with great accuracy. Graph the accelerometer and gyroscope data in two distinct charts as illustrated in Fig. 13, and generate six individual files one for each axis of the accelerometer and gyroscope containing the data in both CSV and MAT formats.

## Limitations

When it comes to the demographics of the people who participated in the dataset, the ratios of women to men (40 % to 60 %), athletes to non-athletes (35 % to 65 %), and left-handed to right-handed people (21 % to 79 %) are satisfactory and acceptable. A diverse age range from 18 to 69 was represented. It is essential to acknowledge that we were unable to achieve uniform percentages for all characteristics among the participants, identify individuals older than the maximum age in this dataset, and include persons with disabilities, specifically upper limb amputees, who wished to participate. Nonetheless, these limits might be regarded as essential goals for initiatives focused on developing new, diversified, and balanced databases in the near future, as they enhance the resources available to researchers in this domain. Nevertheless, challenges were encountered that were deemed exhausting in organizing the sessions for the participants with high accuracy, as a result of the large number of participants and the large number of suggested gestures and their repetitions. Additionally, it was noted that several participants eventually dropped out after one or two sessions, citing special circumstances, boredom with repetitions, or movement difficulties.

## Ethics Statement

Prior to commencing the sessions with the participants, a formal written request for approval was submitted to the Deanship of the College of Engineering at the University of Basrah. The request encompassed a succinct project description, outlining its objectives and the participation of individuals of both genders and various age groups, along with a declaration regarding the participants' objective to construct the dataset while safeguarding their privacy and data confidentiality. The College Dean's Office issued an official letter, coded 7/35/4289, granting approval and authorization on November 20, 2023. Before initiating data collection, informed written consent was acquired from every participant to obtain authorization for processing personal data. As well, all participants received information regarding the study and the nature of the data that will be collected. Participants were afforded the option of either continuing or withdrawing from their participation at any time without additional inquiry. The procedure was conducted in accordance with the ethical standards set forth by the authors' institutions. Moreover, the data is anonymized, ensuring that the identities of the participants remain undisclosed and cannot be inferred from the published information

## CRediT Author Statement

**Khalid A. Abbas:** Conceptualization, Methodology, Software, Validation, Formal analysis, Investigation, Resources, Data Curation, Writing - Original Draft, Visualization. **Mofeed Turky Rashid:** Conceptualization, Writing - Review & Editing, Supervision. **Luigi Fortuna:** Conceptualization, Writing - Review & Editing, Project administration.

## Data Availability

Mendeley DataHand Gesture Accelerometer and Gyroscope Dataset (HGAG-DATA) (Original data).
